# Supplementary material for: Knowledge and Attitudes Concerning Herpes Zoster among People with COPD: An Interventional Survey Study
Source: Vaccines (Basel). 2022 Mar 10;10(3):420. doi: 10.3390/vaccines10030420 (PMC8951084; doi:10.3390/vaccines10030420)
Supplement: Supplementary file 1 [file vaccines-10-00420-s001.zip › vaccines-1607622-supplementary.pdf]

## Supplementary Materials

**Methods S1.** Survey

The survey was conducted online. The survey launched on September 21, 2020, and was open to invited members of the COPD PPRN registry for 7 weeks, closing on November 10, 2020. The estimated time required to complete the survey was 10–15 minutes for Part 1 and 8 minutes for Part 2, including 5 minutes to watch the video (total for Parts 1 and 2: 18–23 minutes). Participants did not see the skip patterns indicated and progressed through the study automatically conditional on their responses.

***Part 1***

Screening question: Do you live in the U.S.?

____Yes [**Proceed**]        _____No [If No, **Exit**]

1. What is your age in years? _____years [If <50, **Exit**]

2. What is your gender?

   ___Male ___Female ___Prefer not to answer ___Other: [Text box_________]

3. How would you rate your overall level of health? *(Please choose one answer)*

   ____Excellent ____ Very Good ____Good ____Fair ____ Poor

4. Thinking about your physical health, which includes COPD and other physical illness or injury, for how many days during the past 30 days was **your physical health not good**? (Enter number of days: 0 to 30)

   _____ days

5. Thinking about your mental health, which includes stress, depression, and problems with emotions, for how many days during the past 30 days was **your mental health not good**? (Enter number of days: 0 to 30)

   _____ days

6. During the past 30 days, how many days did poor physical or mental health keep you **from doing your usual activities**, such as self-care, work or hobbies or play?

(Enter number of days: 0 to 30)

______ days

7. In the past 12 months, have you had any COPD exacerbations OR times when your COPD symptoms worsened, and you were treated with antibiotics or steroid pills?

______Yes [**Go to question 8**] ______No [**Skip to question 9**]

8. If **Yes**, number of episodes ☐☐

9. Have you heard of shingles (also called herpes zoster), a disease causing a blistering skin rash that is often painful and usually in a band-like area on one side of the body?

______Yes [**Go to question 10**] ______No [**Go to question 16**]

10. Have you ever had shingles or herpes zoster?

______Yes, once [**Skip to question 12**]

______Yes, more than once [**Go to question 11**]

______No [**Skip to question 13**]

11. Number of times you have had shingles or herpes zoster? ☐☐

12. When you had shingles, did you experience or do any of the following?
***(Please choose an answer in each line)***

| | No or not very much | Yes, for up to 1 or 2 weeks | Yes, for more than 2 weeks |
|---|---|---|---|
| Skin rash | | | |
| Moderate or severe pain or itching | | | |
| Could not work or do usual activities | | | |
| Trouble sleeping | | | |
| Had to miss social activities | | | |
| Scarring of skin after rash | | | |
| Eye problems from shingles | | | |

| | | | |
|---|---|---|---|
| Increased COPD symptoms | | | |
| Increased shortness of breath | | | |
| Caused a COPD flare or exacerbation | | | |

| | Yes | No |
|---|---|---|
| Needed to see doctor or other health care professional in clinic | | |
| Needed to go to the emergency room | | |
| Was admitted to the hospital | | |
| Went to Urgent Care | | |
| Had to get medicines for the shingles or shingles pain | | |
| Had long lasting pain (more than 3 months) | | |

13. Have any of your family, friends or other people you know ever had shingles (the disease causing a blistering skin rash that is often painful and localized to a band-like area on one side of the body)?

___Yes [**Go to question 14**] ___No [**Skip to question 16**] ___Not sure [**Skip to question 16**]

14. If yes, how many total family members, friends or people you know have had shingles?
_____1 _____2-3 _____ 4-5 _____More than 5 [**Go to question 15**]

15. Thinking of all the people you know who had shingles, did ANY of them have the following problems: ***(Please choose an answer in each line)***

| | Yes | No | I don't know |
|---|---|---|---|
| Skin rash due to shingles | | | |
| Moderate or severe pain or itching due to shingles | | | |
| Could not work or do usual activities due to shingles | | | |
| Trouble sleeping due to shingles | | | |
| Had to miss social activities due to shingles | | | |
| Scarring of skin after rash due to shingles | | | |
| Eye problems from shingles due to shingles | | | |
| Increased COPD symptoms due to shingles | | | |
| Increased shortness of breath due to shingles | | | |
| Caused a COPD flare or exacerbation due to shingles | | | |

| | Yes | No | I don't know |
|---|---|---|---|
| Needed to see doctor or other health care professional in clinic | | | |

| | | | |
|---|---|---|---|
| Need to go to the emergency room | | | |
| Was admitted to the hospital | | | |
| Went to Urgent Care | | | |
| Had to get medicines for the shingles or shingle pain | | | |
| Had long lasting pain (more than 3 months) | | | |

16. How **bothersome** do you think the following are or would be for **people with COPD** who get shingles? (*Choose an answer between 1 to 5 for each line - 1 being not bothersome at all to 5 very bothersome)*

| | 1—not bothersome | 2 | 3 | 4 | 5 —very bothersome |
|---|---|---|---|---|---|
| Shingles rash | | | | | |
| Shingles pain for 1 to 4 weeks | | | | | |
| Shingles pain for more than 4 weeks to 3 months | | | | | |
| Shingles pain for more than 3 months | | | | | |
| Fear of infecting others | | | | | |
| Persistent feelings of sadness or anxiety | | | | | |
| Missing work or usual activities | | | | | |
| Missing social activities | | | | | |
| Increased COPD symptoms | | | | | |
| COPD flare or exacerbation | | | | | |

17. How **important** do you think it is for **people with COPD** to prevent symptoms and problems related to shingles? (*Choose an answer between 1 to 5 for each line - 1 being not important at all to 5 very important)*

| | 1—not important | 2 | 3 | 4 | 5—very important |
|---|---|---|---|---|---|
| Shingles rash | | | | | |
| Shingles pain for 1 to 4 weeks | | | | | |
| Shingles pain for more than 4 weeks to 3 months | | | | | |
| Shingles pain for more than 3 months | | | | | |
| Fear of infecting others | | | | | |
| Persistent feelings of sadness or anxiety | | | | | |
| Missing work or usual activities | | | | | |
| Missing social activities | | | | | |
| Increased COPD symptoms | | | | | |
| COPD flare or exacerbation | | | | | |

18. Compared to other people your age without COPD, what do you think is your risk of getting shingles or getting it again? (choose the best answer for you)

______A lot less risk than other people my age without COPD

______A little less risk at other people my age without COPD

______The same risk as other people my age without COPD

______A little greater risk than someone my age without COPD

______A lot greater risk than someone my age without COPD

______I don't know

19. Do you know that a vaccine or shot is available for preventing shingles in older adults?

______Yes [**Go to question 20**]

______No [**Skip to question 27**]

______Unsure [**Skip to question 27**]

20. How did you hear or learn about the shingles vaccine/shot(s)? If a response does not apply to you check the "Does not apply" box for that line. *(Please choose an answer in each line)*

| Site | Yes | No | Does not apply / Don't remember |
|---|---|---|---|
| At primary care office | | | |
| At pulmonologist's office | | | |
| At a pharmacy | | | |
| At an emergency room visit | | | |
| At an urgent care visit | | | |
| At a hospital stay | | | |
| At a chiropractor's office | | | |
| Information at senior center | | | |
| On Internet | | | |
| TV/Magazine ads | | | |
| Heard from family or friends | | | |

If you heard about the shingles vaccine/shots(s) from another source, please specify.

| |
|---|
| Specify other: [*free text field*] |

21. Have you had either of the shingles vaccine/shot(s)?

______Yes [**Go to question 22**]
______No [**Skip to question 24**]

22. When did you get the shingles vaccine/shot(s)?

______More than 3 years ago
______Within the past 3 years

23. Which kind of the shingles vaccine or shots did you get or have started getting?

______The one-shot kind (Zostavax) [**Skip to question 27**]
______The two-shot kind (Shingrix) [**Skip to question 27**]
______Both kinds [**Skip to question 27**]
______I don't know what kind [**Skip to question 27**]

24. How interested are you in getting the vaccine/shot(s) to prevent shingles (herpes zoster)?

______Undecided [**Go to question 25**]
______Not at all interested [**Go to question 25**]
______Somewhat interested [**Go to question 25**]
______Very interested [**Skip to question 26**]

25. Please share why you are undecided, not at all interested or only somewhat interested in getting the shingles vaccine/shot? *(Please check all that apply)* [**Skip to question 27**]

| | |
|---|---|
| | My doctors/nurse didn't recommend it |
| | No one else suggested I get it |
| | I am not old enough |
| | Costs too much |
| | Worry about side effects |
| | Worry that the shots could give you shingles |
| | Do not think it protects against shingles very well |
| | Do not know enough about the shingles shot |
| | Bad experience with other vaccine |
| | My family/friends had bad experience with shingles shot |
| | My doctor or nurse said I did not need the shingle shot |
| | Have not been able to find a place that has the shingles vaccine to give me |
| | I dislike shots |
| | Not convenient to get shingles shot |
| | Not covered by my insurance |
| | I do not get any vaccines |
| Other reason: [*free text field*] | |

26. Why have you not received the shingles shot(s) even though you are **very interested** in getting it? *(Please check all that apply)*

| | |
|---|---|
| | My doctors/nurse didn't recommend it |
| | I have had the first shot and waiting to get the second one |
| | No one else suggested I get it |
| | I am not old enough |
| | Costs too much |
| | Worry about side effects |
| | Worry that the shots could give me shingles |
| | Not sure it protects against shingles very well |
| | Do not know enough about the shingles shot |
| | Bad experience with other vaccine |
| | My family/friends had bad experience with shingles shot |
| | My doctor or nurse said I did not need the shingle shot |
| | Have not been able to find a place that has the shingles vaccine to give me |
| | I dislike shots |
| | Not convenient to get shingles shot |
| | Not covered by my insurance |
| | I do not get any vaccines |
| Other reason: [*free text field*] | |

27. Has anyone listed below suggested you should get the pneumococcal (sometimes called pneumonia) shot(s)/vaccine(s)? If a question does not apply to you check the "Does not apply" box for that line. *(Choose an answer in each line)*

| Locations | Yes | No | Does not apply / Don't remember |
|---|---|---|---|
| At primary care office | | | |
| At pulmonologist's office | | | |
| At a pharmacy | | | |
| At an emergency room visit | | | |
| At an urgent care visit | | | |
| At a hospital stay | | | |
| At a chiropractor's office | | | |
| Family or friends recommended | | | |

If someone else suggested you should get the pneumococcal shots(s)/vaccine(s), please specify

| |
|---|
| Specify other: [*free text field*] |

28. Has anyone listed below suggested you should get the yearly flu (influenza) shot? If a question does not apply to you check the “Does not apply”” box for that line. *(Choose an answer for each line)*

| Locations | Yes | No | Does not apply/ Don’t remember |
|---|---|---|---|
| At primary care office | | | |
| At pulmonologist’s office | | | |
| At a pharmacy | | | |
| At an emergency room visit | | | |
| At an urgent care visit | | | |
| At a hospital stay | | | |
| At a chiropractor’s office | | | |
| Family or friends recommended | | | |

If someone else suggested you should get the yearly flu (influenza) shot, please specify

| |
|---|
| Specify other: [*free text field*] |

29. Has the COVID-19 pandemic changed your interest in getting any of these shots or vaccines? *(Choose an answer for each line)* [**Part 1 completed**]

| | Yes, increased interest | Yes, decreased interest | No change |
|---|---|---|---|
| Shingles vaccine | | | |
| Influenza vaccine | | | |
| Pneumococcal/pneumonia vaccine | | | |

- **Finish for those who answered they have received or started the two-shot HZ vaccine (Shingrix), responding “The two-shot kind (Shingrix)” or “Both kinds” to Q23. [Exit]**

- **Continue to Part 2 and the post-video questions if <u>any</u> of the following questions were answered with the indicated response(s):**
  **Q9 – “No”**
  **Q18 – “A lot less risk”, “A little less risk”, “The same risk” or “I don’t know”**
  **Q19 – “No” or “Unsure”**
  **Q21 – “No”**
  **Q23 – “The one-shot kind (Zostavax)” or “I don’t know what kind”**
  **[Go to question 30]**

***<u>Part 2</u>***

**Please watch the following 5-minute video on COPD and herpes zoster and answer the questions after completing the video.**

**Embedded video**

**Questions presented after video:**

30. How useful was the information in the video about: *(Choose an answer for each line)*

| | Not useful at all | Not very useful | Not sure | A little useful | Very useful |
|---|---|---|---|---|---|
| General information on the risk and burden of herpes zoster/shingles? | | | | | |
| Increased risk of shingles/herpes zoster in people with COPD? | | | | | |
| Complications of herpes zoster/shingles? | | | | | |
| Who to ask about herpes zoster/shingles shots? | | | | | |
| Recommendations for who should get the shingles/herpes zoster shots? | | | | | |

31. Based on the information in the video, how does having COPD affect your risk of getting shingles?

___Decreases the risk
____Does not change the risk
____Increases the risk
____I am still not sure

32. After watching the video, has your level of interest in getting the shingles/herpes zoster shots changed?

_____Less interested [**Go to question 33**]
_____No change [**Skip to question 34**]
_____More interested [**Skip to question 34**]

33. Why are you less interested?

[*Free text field*] [**Exit**]

34. Do you now plan to get the shingles vaccine?

_____Yes, plan to get the shingles vaccine [**Exit**]
_____Not sure but will talk to doctor or nurse [**Exit**]
_____Not sure but will consider [**Exit**]
_____No do not plan to get the shingles vaccine [**Go to question 35**]

35. Why will you not get the shingles vaccine?

[*Free text field*] [**Exit**]

[Survey end]

**Methods S2.** Educational video intervention

The survey included an intervention phase in the form of a short (five-minute) educational video on the epidemiology, risk, burden, impact, and opportunities for HZ prevention through vaccination. The educational video can be viewed at: ShiPPS Patient Video. Conditional on their answers to questions in Part 1 of the survey, participants who had not heard of HZ or HZ vaccines, those who had not received or started the two-shot HZ vaccine (Shingrix), and those who were unaware of the increased risk of HZ for people with COPD, were directed to and watched the video.

## Supplementary Tables

**Table S1.** Associations between increased rates of COPD-related symptoms and baseline characteristics

| | Increased COPD symptoms, % (n) | | | Increased dyspnea, % (n) | | | Increased exacerbations, % (n) | | |
|---|---|---|---|---|---|---|---|---|---|
| **Baseline characteristic** | **Yes** | **No** | **p-value*** | **Yes** | **No** | **p-value*** | **Yes** | **No** | **p-value*** |
| ***Poor physical health*** | | | | | | | | | |
| 0–5 days/month (n=79) | 7.6 (6) | 92.4 (73) | | 11.4 (9) | 88.6 (70) | | 3.8 (3) | 96.2 (76) | |
| 6–15 days/month (n=45) | 24.4 (11) | 75.6 (34) | 0.002 | 35.6 (16) | 64.4 (29) | <0.001 | 17.8 (8) | 82.2 (37) | 0.006 |
| 16–30 days/month (n=68) | 27.9 (19) | 72.1 (49) | | 35.3 (24) | 64.7 (44) | | 17.6 (12) | 82.4 (56) | |
| ***Poor mental health*** | | | | | | | | | |
| 0–5 days/month (n=121) | 13.2 (16) | 86.8 (105) | | 19.8 (24) | 80.2 (97) | | 6.6 (8) | 93.4 (113) | |
| 6–15 days/month (n=40) | 25.0 (10) | 75.0 (30) | 0.059 | 27.5 (11) | 72.5 (29) | 0.038 | 20.0 (8) | 80.0 (32) | 0.035 |
| 16–30 days/month (n=31) | 32.3 (10) | 67.7 (21) | | 45.2 (14) | 54.8 (17) | | 22.6 (7) | 77.4 (24) | |
| ***Unable to work/do usual activities*** | | | | | | | | | |
| 0–5 days/month (n=99) | 10.0 (10) | 89.9 (89) | | 14.1 (14) | 85.9 (85) | | 5.1 (5) | 94.9 (94) | |
| 6–15 days/month (n=41) | 29.3 (12) | 70.7 (29) | 0.006 | 41.5 (17) | 58.5 (24) | 0.001 | 19.5 (8) | 80.5 (33) | 0.013 |
| 16–30 days/month (n=52) | 26.9 (14) | 73.1 (38) | | 34.6 (18) | 65.4 (34) | | 19.2 (10) | 80.8 (42) | |
| ***Exacerbations in prior 12 months*** | | | | | | | | | |
| 0 episodes (n=76) | 10.5 (8) | 89.5 (68) | | 14.5 (11) | 85.5 (65) | | 3.9 (3) | 96.1 (79) | |
| 1 episode (n=38) | 10.5 (4) | 89.5 (34) | 0.005 | 21.1 (8) | 78.9 (30) | 0.003 | 5.3 (2) | 94.7 (36) | 0.002 |
| 2+ episodes (n=78) | 30.8 (24) | 69.2 (54) | | 38.5 (30) | 61.5 (48) | | 23.1 (18) | 76.9 (60) | |

*P-values derived from Welch's F-Test comparing the frequency of participants experiencing increased symptoms across the three indicated groups for each baseline characteristic.

*Abbreviations:* COPD, chronic obstructive pulmonary disease.

**Table S2.** Participants' reporting of the symptoms and problems experienced by the people they knew who had experienced HZ (N=492*)

| Symptom/Problem† | Yes, % (n) | No, % (n) | Do not remember/ Do not know, % (n) |
|---|---|---|---|
| Skin rash | 93.1 (458) | 2.0 (10) | 4.9 (24) |
| Moderate/severe pain or itching | 94.9 (467) | 1.0 (5) | 4.1 (20) |
| Scarring of skin | 25.6 (126) | 20.9 (103) | 53.5 (263) |
| Unable to work/do usual activities | 61.8 (304) | 14.4 (71) | 23.8 (117) |
| Missed social activities | 60.4 (297) | 7.1 (35) | 32.5 (160) |
| Trouble sleeping | 66.7 (328) | 3.9 (19) | 29.5 (145) |
| Eye problems | 15.9 (78) | 33.3 (164) | 50.8 (250) |
| COPD: Increased symptoms | 5.5 (27) | 42.5 (209) | 52.0 (256) |
| COPD: Shortness of breath | 9.1 (45) | 35.6 (175) | 55.3 (272) |
| COPD: Exacerbation | 5.1 (25) | 41.3 (203) | 53.7 (264) |
| Visited physician/HCP in clinic | 86.4 (425) | 4.5 (22) | 9.1 (45) |
| Required medication for HZ/pain | 83.3 (410) | 2.0 (10) | 14.6 (72) |
| Long-lasting pain (>3 months) | 38.0 (187) | 23.2 (114) | 38.8 (191) |

*Participants who responded that they knew at least one other person who had had HZ.

†A response was required for each of the listed symptoms/problems.

*Abbreviations:* COPD, chronic obstructive pulmonary disease; HCP, healthcare professional; HZ, herpes zoster.

**Table S3.** Percentage of participants who reported that they remember discussion about or receiving recommendations for specific vaccines at the locations they have attended

| Location/Source* | Herpes zoster immunization % Yes, (n) | "Pneumonia" immunization % Yes, (n) | Influenza immunization % Yes, (n) |
|---|---|---|---|
| At primary care office | 68.8 (425) | 88.7 (618) | 94.4 (670) |
| At pulmonologist's office | 26.6 (149) | 71.0 (450) | 75.1 (489) |
| At a pharmacy | 48.5 (283) | 36.7 (217) | 60.9 (389) |
| At a hospital stay | 5.2 (25) | 23.1 (118) | 24.9 (123) |
| On the internet | 42.1 (236) | NA | NA |
| TV/Magazine advertisement | 50.8 (287) | NA | NA |
| Family or friends | 55.3 (330) | 29.5 (166) | 44.8 (267) |

*The total N for each location or source is based on those who answered the question and did not respond that the location/source did not apply or that they did not remember.

*Abbreviations:* NA, not asked; TV, television.

## Supplementary Figures

**Figure S1.** Most frequently reported barriers to participants with COPD getting the HZ vaccination

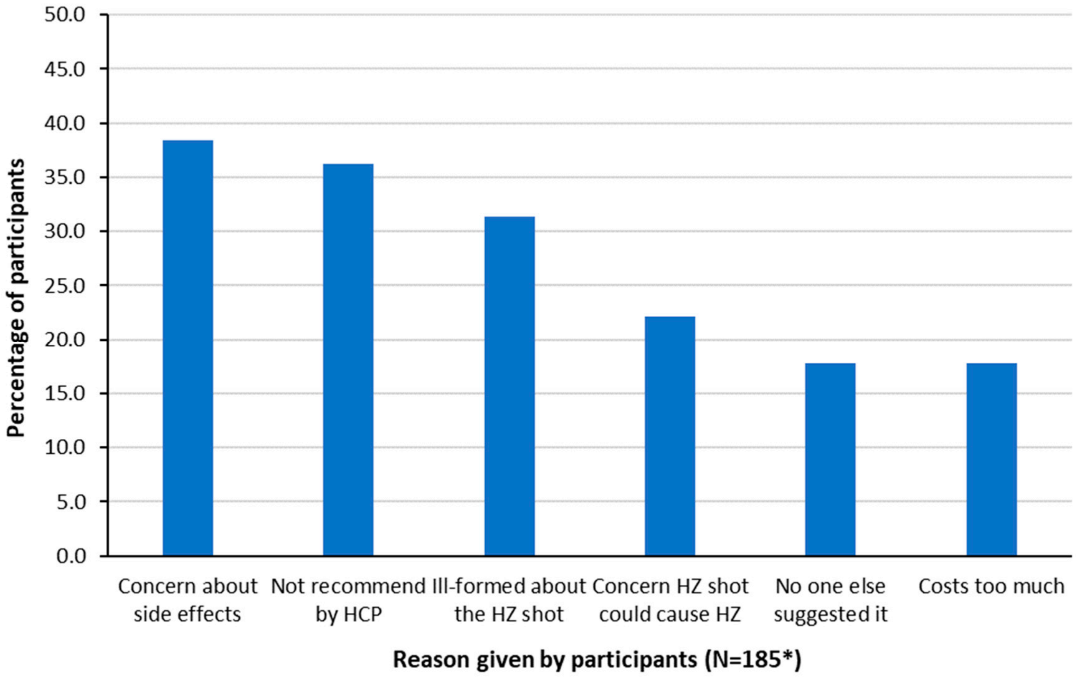


Shown are the five most common reasons that participants with COPD were undecided, not interested or only somewhat interested in getting the HZ vaccine.

*N=185 participants who were unvaccinated and responded they were undecided, not interested, or only somewhat interested in vaccination.

*Abbreviations:* COPD, chronic obstructive pulmonary disease; HCP, healthcare professional; HZ, herpes zoster.
